# Indicators for prediction of *Mycobacterium tuberculosis* positivity detected with bronchoalveolar lavage fluid

**DOI:** 10.1186/s40249-018-0403-x

**Published:** 2018-03-24

**Authors:** Xi Liu, Xing-Fang Hou, Lei Gao, Guo-Fang Deng, Ming-Xia Zhang, Qun-Yi Deng, Tao-Sheng Ye, Qian-Ting Yang, Bo-Ping Zhou, Zhi-Hua Wen, Hai-Ying Liu, Hardy Kornfeld, Xin-Chun Chen

**Affiliations:** 1 Shenzhen Key Laboratory of Infection & Immunity, Shenzhen Third People’s Hospital (The Second Affiliated Hospital of Shenzhen University), Shenzhen University School of Medicine, Shenzhen, China; 2 Department of Infectious Diseases, the Fifth Affiliated Hospital, Sun Yat-sen University, Zhuhai, China; 3 Ministry of Health Key Laboratory of Systems Biology of Pathogens, Institute of Pathogen Biology, Chinese Academy of Medical Sciences and Peking Union Medical College, Beijing, China; 4 Department of Medicine, University of Massachusetts Medical School, Worcester, MA 01655 USA; 5 Yuebei Second People’s Hospital, Shaoguan, China; 6 Department of Pathogen Biology, Shenzhen University School of Medicine, Shenzhen, 518054 China

**Keywords:** Pulmonary tuberculosis, Predictive factors, Bronchoalveolar lavage fluid, *Mycobacterium tuberculosis*, Detection

## Abstract

**Background:**

The diagnosis of active pulmonary tuberculosis (TB) remains a challenge in clinic, especially for sputum negative pulmonary TB. Bronchoalveolar lavage fluid (BALF) has higher sensitivity than sputum for detection of *Mycobacterium tuberculosis* (Mtb). However, bronchoscopy is invasive and costly, and not suitable for all patients. In order to make TB patients get more benefit from BALF for diagnosis, we explore which indicator might be used to optimize the choice of bronchoscopy.

**Methods:**

A total of 1539 sputum-smear-negative pulmonary TB suspects who underwent bronchoscopy were recruited for evaluation. The sensitivity, specificity and accuracy of Mtb detection in sputum and BALF were compared. Odds ratios and 95% confidence intervals were used to assess variables that associated with positive acid-fast bacilli (AFB) smear, Mtb culture and nucleic acid amplification test (NAAT) of BALF in sputum-negative and non-sputum-producing pulmonary TB suspects.

**Results:**

BALF has significantly higher sensitivity (63.4%) than sputum (43.5%) for Mtb detection by culture and NAAT. 19.7% (122/620) sputum-negative and 40.0% (163/408) non-sputum-producing suspects had positive bacteriological results in BALF. Among sputum-negative and non-sputum-producing pulmonary TB suspects, the positivity of Mtb detection in BALF is associated with a younger age, the presence of pulmonary cavities and a positive result of interferon-gamma release assay (IGRA). Sputum-negative patients under 35 years old with positive IGRA and pulmonary cavity had 84.8% positivity of Mtb in BALF.

**Conclusions:**

Our study indicated that combination of age, the presence of pulmonary cavity, and the result of IGRA is useful to predict the positivity of Mtb detection in BALF among sputum-negative and non-sputum producing pulmonary TB suspects. Those who are under 35 years old, positive for the presence of pulmonary cavity and IGRA, should undergo bronchoscopy to collect BAFL for Mtb tests, as they have the highest possibility to get bacteriologically confirmation of TB.

**Electronic supplementary material:**

The online version of this article (10.1186/s40249-018-0403-x) contains supplementary material, which is available to authorized users.

## Multilingual abstracts

Please see Additional file 1 for translations of the abstract into the five official working languages of the United Nations.

## Background

Tuberculosis (TB), an airborne infection caused by *Mycobacterium tuberculosis* (Mtb), remains a major global health problem. In 2015, there were estimated 10.4 million new TB cases, and 1.8 million TB deaths. Despite this large toll of mortality, majority of TB patients can be cured within 6 months with timely diagnosis and correct treatment [1]. Considering there is no effective preventive vaccine against TB, the early diagnosis of pulmonary TB patients is not only critical for individual treatment, but also a major strategy for prevention of TB transmission. Microbiological confirmation of TB and drug susceptibility testing are key factors ensuring that new TB suspects are accurately diagnosed and timely accessible to effective treatment. Unfortunately, it remains as a challenge as up to 65% of TB patients were negative for Mtb detection in China [1]. There is a clear necessity to identity optimal methods for pulmonary TB diagnosis of bacteriologically-negative patients, particularly in high burden settings of multi-drug resistant TB (MDR-TB) like China.

Newer methods with increased sensitivity compared to traditional acid-fast bacilli (AFB) smear and culture have been developed during the last decades [2]. For example, molecular DNA-based diagnostics such as Xpert MTB/RIF [3] and line probe assays (LPAs) [4], have become widely available and permit both rapid diagnosis and preliminary assessment of drug susceptibility [5]. While by smear, culture and nucleic acid amplification test (NAATs) have made an important contribution by accelerating TB diagnosis and preliminary identification of MDR-TB, the sensitivity remains too low [6]. Consequently, a substantial proportion of pulmonary TB patients are diagnosed based only on symptoms, chest X-ray abnormalities or (rarely) characteristic histopathology [7]. In China, the positive rate of bacteriologically confirmed pulmonary TB is only 35% among 1.38 million pulmonary TB cases [1]. Among these patients, some were bacteriologically negative due to lack of sputum or false-negative due to poor sputum sample quality. Bronchoscopy has demonstrated superiority to expectorated sputum for pulmonary TB diagnosis. As an example, a recent study reported that 54% of sputum-negative pulmonary TB patients were eventually confirmed by finding Mtb in BALF samples by NAAT or culture [8].

Although BALF offers the potential to improve the positivity of microbiological finding for diagnosis of pulmonary TB, bronchoscopy is invasive and costly. In order to maximize the benefit of bronchoscopy while minimizing the cost to TB control programs, it is important to identify parameters to predict the yield of BALF for diagnosis of sputum- negative pulmonary TB [9]. In the present study, we compared the sensitivity and specificity of Mtb tests between sputum and BALF for diagnosis of pulmonary TB among 1539 sputum-smear-negative cases, and identified clinical parameters which are associated with the benefit of bronchoscopy in sputum-negative and non-sputum-producing pulmonary TB suspects.

## Methods

### Study population

This retrospective study was conducted at Shenzhen Third People’s Hospital (Guangdong, China). Between February 2011 and May 2015, a total of 1539 sputum-smear-negative pulmonary TB suspects who underwent bronchoscopy for medical diagnosis purpose were included in the study. The selection of TB suspects for bronchoscopy was made at the discretion of the clinic physicians responsible for their care with no influence from the investigators implementing this study. Medical data were collected on age, gender, symptoms, previous TB history, laboratory test and imaging detection. Laboratory tests included sputum smear (three times) and culture, interferon-gamma release assay (IGRA), CD4 and CD8 cell counts. Patients with a follow-up less than 6 months were excluded.

### Cases definition

The diagnosis of active TB were following Chinese diagnostic criteria for tuberculosis (WS288–2008), WHO guidelines for treatment of tuberculosis (fourth edition), and other references [10–12]. Active TB cases were classified as definite TB and probable TB, the definition and category of study population were listed below:

Tuberculosis suspect referred to any person who presented with typical chest imaging suggestive of TB (WS288–2008).

Definite TB was defined as sputum or BALF culture positive for Mtb, or NAAT positive for Mtb, plus clinical symptom and CT sign suggestive of pulmonary TB and response to anti-tuberculosis treatment.

Probable TB was defined as sputum or BALF culture and NAAT negative for Mtb, sputum/BALF negative or positive for AFB, plus clinical symptom and CT sign suggestive of pulmonary TB and response to anti-tuberculosis treatment.

Cured TB (RxTB) was defined as the pulmonary TB patients who had completed standard anti-TB treatment regimen for at least 6 months with no subsequent evidence of active TB.

Non-TB lung disease (non-TB) was defined as negative cultures and smears for Mtb detection in sputum and BALF, CT suggestive of non-TB, and anti-TB treatment was never initiated by healthcare providers. Non-TB lung diseases included bacterial pneumonia, lung cancer, sarcoidosis, asthma, bronchiectasis, bronchitis, etc.

Non-tuberculous mycobacteria (NTM) infection was confirmed by identification of the same species of NTM in sputum and/or BALF.

### Bronchoscopy technique, specimen processing and IGRA

All patients underwent bronchoscopy with BALF collection. Patients were pre-medicated with 0.5 mg atropine and then sedated with midazolam. Bronchial washing of the involved sub-segment was performed with 20 to 50 ml of isotonic saline. Sputum samples, if available, were collected on three consecutive days and examined for the presence of acid-fast bacilli by microscopy. All sputum and BALF samples were subjected to NAAT for detection of Mtb DNA using a commercial quantitative real-time polymerase chain reaction kit (qPCR) (Qiagen, China), and mycobacterium culture for up to 6 weeks in fluid media (MGIT, BD, Heidelberg), or 8 weeks in solid media (Loewenstein-Jensen), before the results were concluded negative.

The Mtb-specific IGRA was performed as an in-house IFN-γ enzyme-linked immunospot assay which was reported previously [13]. The overall sensitivities and specificities of our in-house IGRA were similar to commercial IGRA kits [13].

### Statistical analysis

The positive rate, sensitivity, specificity and accuracy of Mtb tests in sputum and BALF for the diagnosis of pulmonary TB were calculated.

For categorical variables, the percentage of patients in each category was calculated. To identify potential variables related to positivity of BALF (positivity in Mtb detection of BALF through smear microscopy, culture and/or NAAT), univariate analysis with Pearson’s *χ*^2^ test was performed. All variables with *P* values less than 0.05 in univariate analysis were entered into the unconditional multiple logistic regression analyses and the associations assessed with Odds ratios (*OR*) and 95% confidence intervals (*CI*s). To identify the overlapping effects on positivity of BALF, variables with *P* values less than 0.05 in unconditional multiple logistic regression analyses were entered into stratified analysis. All analyses were performed using SPSS software (release 20.0, IBM,USA).

## Results

### Clinical characteristics and final diagnosis of sputum-smear-negative pulmonary TB suspects

**Fig. 1:**
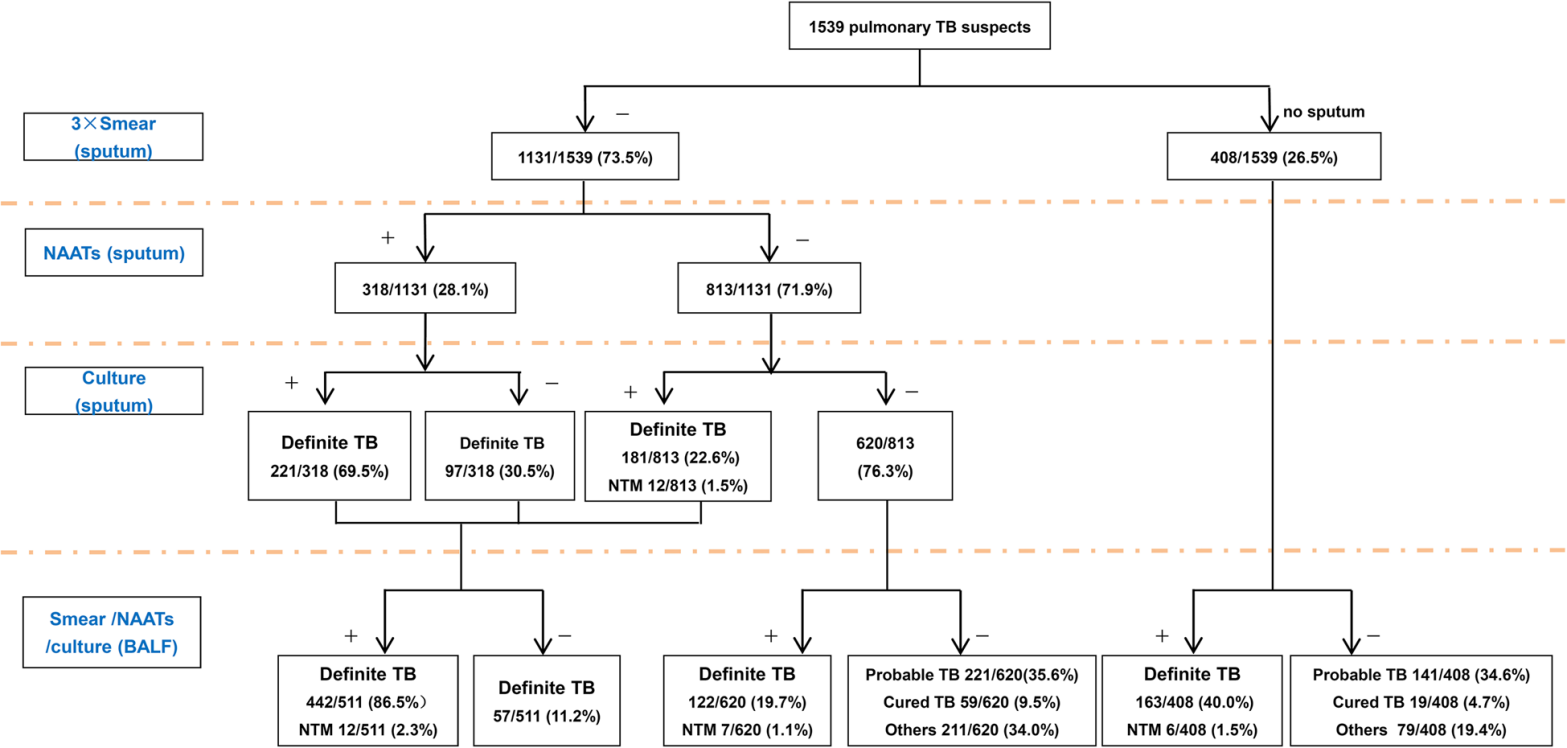
Diagnostic flow chart. Abbreviations: NTM, nontuberculosis mycobateria; NAAT, nucleic acid amplification technique; BALF, bronchoalveolar lavage fluid

A total of 1539 subjects who underwent bronchoscopy were enrolled in the study (see Fig. 1). The median age was 36.8 ± 13.9 (interquartile range, 11 to 81 years old) and 56.2% were males. There were no complications from the procedure and BALF samples were obtained from all patients. Among them, 1146 patients had a final diagnosis of pulmonary TB, 78 patients were cured TB but not TB relapse, 25 patients were diagnosed with NTM infection, and 290 patients had a final diagnosis of non-TB lung diseases (see Table 1).

**Table 1:** Clinical characters of pulmonary TB suspects who underwent bronchoscopy

Final diagnosis	Number	Age (mean ± *SD*)	Male (%)
Active pulmonary TB	1146	35.0 ± 13.5	636 (55.5%)
Cured tuberculosis	78	42.9 ± 13.3	51 (65.4%)
NTM	25	45.5 ± 15.9	8 (32.0%)
Other lung disease	290	41.4 ± 15.1	170 (58.6%)
Pneumonia	193	40.9 ± 15.9	111 (57.5%)
Lung cancer	34	49.8 ± 13.9	21 (61.8%)
Others^a^	62	38.4 ± 11.5	38 (61.3%)
Total	1539	36.8 ± 13.9	865 (56.2%)

*TB* tuberculosis, *NTM* nontuberculosis mycobateria

^a^Others included sarcoidosis, asthma, bronchiectasis, bronchitis, etc

Among 1539 suspect TB patients, 620 (40.3%) patients were negative for sputum Mtb culture or NAAT test, 408 (26.5%) patients had no sputum. Of 1146 suspects with final diagnosis of pulmonary TB, 304 (26.5%) had no sputum production, 343 (29.9%) were negative for sputum Mtb detection by smear, culture or NAAT, while 499 (43.5%) were bacteriologically confirmed based on one or more of these sputum tests. In comparison, 727 of 1146 (63.4%) pulmonary TB patients were bacteriologically confirmed by smear, culture or NAAT in BALF. A total of 362 (31.6%) patients had a final clinical diagnosis of pulmonary TB based on chest imaging and anti-TB treatment response.

Among 25 NTM lung disease cases who were diagnosed by conventional mycobacterial culture and 16 s RNA gene based molecular species identification using BALF samples, only 48.0% (12/25) cases were identified by sputum culture. Bronchoscopy made more species identification possible, from which a correct treatment could be applied.

### BALF improved the diagnostic accuracy of sputum-negative and non-sputum-producing TB suspects

Positive detectable rate, sensitivity, specificity and accuracy of different methods for the diagnosis of all sputum-smear-negative pulmonary TB suspects, sputum negative and no sputum patients were listed in Tables 2 and 3. Among the 1539 suspects, the overall positive detection rate of BALF for diagnosis of pulmonary TB was significantly higher than that of sputum, with overall positivity of 47.2% and 32.4%, respectively. The overall sensitivity of BALF (63.4%) was significantly higher than that of sputum (43.5%) (see Table 2). Compared to sputum, an increased sensitivity of BALF was observed in all three Mtb tests including AFB staining, Mtb culture and NAAT, indicating that BALF samples were enriched for Mtb. Specifically, 122 (19.7%) out of 620 pulmonary TB patients who were negative for all three Mtb tests in sputum were eventually confirmed by positive results for one or more of these tests in BALF. Notably, the benefit of BALF was mostly evident in those pulmonary TB suspects without sputum production. As shown in Table 3, 163 (40.0%) out of 408 pulmonary TB patients without sputum production were bacteriologically confirmed by BALF Mtb tests.

**Table 2:** Comparison of sensitivity, specificity, etc. between sputum and BALF for the diagnosis of pulmonary TB in sputum-smear-negative suspects

	Positive detectable rate (%)	Sensitivity (%)	Specificity (%)	Accuracy (%)
Sputum-culture/NAAT	32.4	43.5	100.0	58.0
sputum-culture	26.1	35.1	100.0	51.7
sputum-NAAT	20.7	27.7	100.0	46.2
BALF-smear/culture/NAAT	47.2	63.4	100.0	72.8
BALF-smear	9.2	12.0	99.2	34.3
BALF-culture	42.5	57.1	100.0	68.0
BALF-NAAT	33.2	44.5	99.7	58.6
Sputum+BALF	50.9	68.4	100.0	72.8

*TB* tuberculosis, *BALF* bronchoalveolar lavage fluid, *NAAT* nucleic acid amplification test

**Table 3:** The performance of BALF based detection for the diagnosis of pulmonary TB in sputum-negative suspects and those without sputum

	Positive detectable rate %	Sensitivity %	Specificity %	Accuracy %
Sputum-negative				
BALF-smear/culture/NAAT	19.7	35.6	100.0	64.4
BALF-culture	16.5	29.7	100.0	61.1
BALF-NAAT	11.1	20.1	100.0	55.8
No sputum				
BALF-smear/culture/NAAT	40.0	53.6	100.0	65.4
BALF-culture	38.2	51.3	100.0	63.7
BALF-NAAT	24.5	32.9	100.0	50.0
sputum negative and no sputum				
BALF-smear/culture/NAAT	27.8	44.1	100.0	64.9
BALF-culture	26.3	41.6	100.0	63.3
BALF-NAAT	16.5	26.3	100.0	53.7

*TB* tuberculosis, *BALF* bronchoalveolar lavage fluid, *NAAT* nucleic acid amplification test

### Factors associated with positive BALF Mtb detection in sputum-negative and non-sputum-producing pulmonary TB patients

In line with previous literature [14], our data demonstrated that BALF significantly improved Mtb detection in pulmonary TB patients. More importantly, BALF significantly improved the diagnosis and treatment of those pulmonary TB patients who were unable to produce sputum. Isolating Mtb in those cases was not only critical for TB diagnosis, but also permitted drug susceptibility testing. Despite the clear benefit of bronchoscopy in the management of individual cases, only 285 (27.7%) subjects were positive for Mtb detection in BALF among 1028 subjects with sputum negative or without sputum. To identify those patients most likely to benefit from bronchoscopy, we analyzed the clinical parameters associated with Mtb detection in BALF among sputum-negative pulmonary TB suspects and TB suspects who were unable to produce sputum. Univariate analysis showed that age (*P* <  0.001), hemoptysis (*P* = 0.002), bilateral lung infection (*P* = 0.005), cavity on chest radiographs (*P* <  0.001), CD4 cell count (*P* = 0.004), CD8 cell count (*P* = 0.001), erythrocyte sedimentation rate (*P* = 0.001), and IGRA results (*P* <  0.001) were all significantly associated with Mtb detection in BALF (see Table 4). Multivariate logistic regression analysis revealed that age, cavity on chest radiographs (*OR*: 4.108, 95% *CI*: 2.376–7.102), IGRA result (*OR*: 3.743, 95% *CI*: 2.478–5.655), and CD8 counts (*OR*: 1.992, 95% *CI*: 1.289–3.076) were independent risk factors associated with the positivity of Mtb detection in BALF (see Table 5). Further analysis indicated that combination of age, cavity on chest radiographs, and IGRA yielded the highest positivity of Mtb detection in BALF. Specifically, younger pulmonary TB suspects (**≤** 35 years old) with cavity on chest radiographs and positive IGRA had 84.8% (28/33) positivity of Mtb detection in BALF (see Table 6).

**Table 4:** Comparison of clinical characteristics between BALF bacteriologically negative and positive patients within sputum negative pulmonary TB patients and those without sputum

	Total	BALF (+)	BALF (−)	*P*-value	*OR* (95% *CI*)
	*N* = 1028	*n* = 285	*n* = 743		
Age	37.4 ± 14.2	32.7 ± 12.8	39.3 ± 14.2	< 0.001	
Male	589	154 (26.1%)	435 (73.9%)	0.191	0.832 (0.632~ 1.096)
TB history	126	40 (31.7%)	86 (68.3%)	0.282	1.247 (0.834–1.866)
Fever	363	94 (25.9%)	269(74.1%)	0.333	0.867 (0.650–1.157)
Night sweat	97	28 (28.9%)	69 (71.1%)	0.792	1.064 (0.670–1.689)
Cough	751	213 (28.4%)	538 (71.6%)	0.451	1.127 (0.825–1.540)
Hemoptysis	158	60 (38.0%)	98 (62.0%)	0.002	1.755 (1.230–2.504)
Lose weight	222	72 (32.4%)	150 (67.6%)	0.077	1.336 (0.969–1.843)
Bilateral lung	268	92 (34.3%)	176 (65.7%)	0.005	1.536 (1.137–2.074)
Cavity	104	57 (54.8%)	47 (45.2%)	< 0.001	4.108 (2.376–7.102)
^a^CD4	595.8 ± 274.4	547.77 ± 246.14	615.8 ± 283.2	0.004	
^a^CD4≤500	264	92 (34.8%)	172 (65.2%)	0.011	1.540 (1.101–2.153)
^a^CD8	420.0 ± 205.4	380.30 ± 181.83	436.7 ± 212.6	0.001	
^a^CD8≤500	468	153 (32.7%)	315 (67.3%)	0.002	1.992 (1.289–3.076)
ESR	40.9 ± 31.3	46.08 ± 29.97	39.0 ± 31.6	0.001	
IGRA (+)^b^	507	201 (39.6%)	306 (60.4%)	< 0.001	3.743 (2.478–5.655)

*BALF* bronchoalveolar lavage fluid, *IGRA* interferon-γ release assays, *ESR* erythrocyte sedimentation rate

^a^Total *N* = 679, BALF (+) *n* = 199, BALF (−) *n* = 480

^b^Total *N* = 938, BALF (+) *n* = 270, BALF (−) *n* = 692

**Table 5:** Univariate and multivariate analysis of factors associated with BALF positivity in pulmonary TB sputum-negative suspects and those without sputum

	Total	BALF (+)	BALF (−)	*P*-value	*OR* (95% *CI*)^a^
		*n* = 285 (%)	*n* = 743 (%)		
Age (year)				< 0.001	
≤ 25	244	105 (43.0)	139 (57.0)		Reference
25–35	288	88 (30.6)	200 (69.4)		0.582 (0.408–0.832)
35–45	215	44 (20.5)	171 (79.5)		0.341 (0.224–0.517)
45–55	128	22 (17.2)	116 (82.8)		0.251 (0.149–0.423)
55–65	106	23 (21.7)	83 (78.3)		0.367 (0.217–0.621)
> 65	37	3 (8.1)	34 (91.9)		0.117 (0.035–0.391)
Cavity				< 0.001	
No	924	228 (24.7)	696 (75.3)		Reference
Yes	104	57 (54.8)	47 (45.2)		4.108 (2.376–7.102)
IGRA				< 0.001	
Negative	431	64 (14.8)	367 (85.2)		Reference
Positive	507	201 (39.6)	306 (60.4)		3.743 (2.478–5.655)
CD8+				0.002	
> 500	205	46 (22.5)	159 (77.5)		Reference
≤ 500	468	153 (32.7)	315 (67.3)		1.992 (1.289–3.076)

*TB* tuberculosis, *BALF* bronchoalveolar lavage fluid, *NAAT* nucleic acid amplification test, *IGRA* interferon-γ release assays

^a^Controlling for variables with *P* < 0.05 in univariate analysis

**Table 6:** Stratified analysis of factors associated with BALF positivity in pulmonary TB sputum-negative suspects and those without sputum

	BALF (+) *n/N* (%)	BALF (−) *n/N* (%)	*P*-value	*OR* (95% *CI*)
IGRA/Age			<0.001	
IGRA (−)/> 35 year	30/239 (12.6)	209/239 (87.4)		Reference
IGRA (−)/≤ 35 year	34/192 (17.7)	158/192 (82.3)		1.499 (0.880–2.554)
IGRA (+)/> 35 year	55/212 (25.9)	157/212 (74.1)		2.441 (1.494–3.986)
IGRA(+)/≤ 35 year	146/295 (49.5)	149/295 (50.5)		6.826 (4.372–10.658)
Cavity/Age			<0.001	
Cavity (−)/> 35 year	76/453 (17.0)	371/477 (83.0)		Reference
Cavity (−)/≤ 35 year	152/477 (31.9)	325/477 (68.1)		2.283 (1.669–3.122)
Cavity (+)/> 35 year	16/49 (32.7)	33/49 (67.3)		2.367 (1.240–4.516)
Cavity (+)/≤ 35 year	41/55 (74.5)	14/55 (25.5)		14.296 (7.426–27.521)
CD8/Age			<0.001	
CD8> 500/> 35 year	16/82 (19.5)	66/82 (80.5)		Reference
CD8> 500/≤ 35 year	30/117 (25.6)	87/117 (74.4)		1.422 (0.716–2.824)
CD8 ≤ 500/> 35 year	49/222 (22.1)	173/222 (77.9)		1.168 (0.621–2.197)
CD8 ≤ 500/ ≤ 35 year	104/246 (42.3)	142/246 (57.7)		3.021 (1.655–5.515)
IGRA/Cavity/Age			<0.001	
IGRA (−)/Cavity (−)/> 35 year	24/219 (11.0)	195/219 (89.0)		Reference
IGRA (−)/Cavity (−)/≤ 35 year	23/174 (13.2)	151/174 (86.8)		1.238 (0.672–2.278)
IGRA (−)/Cavity (+)/> 35 year	6/20 (30.0)	14/20 (70.0)		3.482 (1.223–9.912)
IGRA (−)/Cavity (+)/≤ 35 year	11/18 (61.1)	7/18 (38.9)		12.768 (4.521–36.056)
IGRA (+)/Cavity (−)/> 35 year	45/184 (24.5)	139/184 (75.5)		2.630 (1.531–4.519)
IGRA (+)/Cavity (−)/≤ 35 year	118/262 (45.0)	144/262 (55.0)		6.658 (4.083–10.856)
IGRA (+)/Cavity (+)/> 35 year	10/28 (35.7)	18/28 (64.3)		4.514 (1.869–10.901)
IGRA (+)/Cavity (+)/≤ 35 year	28/33 (84.8)	5/33 (15.2)		45.500 (16.054–128.955)

*TB* tuberculosis, *BALF* bronchoalveolar lavage fluid, *IGRA* interferon-γ release assays

## Discussion

Negative diagnostic tests on sputum samples and lack of sputum production in patients with pulmonary TB have been reported to be the major causes of treatment delay [15]. In our study, the sensitivity of sputum Mtb tests including sputum Mtb culture and NAAT was 43.5% (499/1146) in pulmonary TB patients. In paired comparison, 63.4% of (727/1146) pulmonary TB patients were bacteriologically confirmed by BALF Mtb tests. In addition, 13 out of 25 patients with NTM infection were diagnosed by BALF-based tests. Thus, about 30% patients benefited from BALF Mtb tests. It is worthy to note that BALF Mtb tests increased 35.6% and 53.6% positivity in sputum-negative and non-sputum-producing pulmonary TB patients, respectively. BALF not only increased the positive rate of bacteriological detection for diagnosis, but also made a significant improvement for effective precision treatment through enabling species identification and drug susceptibility testing.

While bronchoscopy is clearly a useful diagnostic tool for pulmonary TB, it has risks of hemorrhage, pneumothorax, laryngospasm as well as other minor and major potentially adverse effects [16, 17]. Nosocomial transmission of TB and other pathogens might also occur in some patients exposed to inadequately sterilized bronchoscopy equipment. Furthermore, bronchoscopy is an expensive procedure requiring expert personnel and facilities. Therefore, we identified clinical parameters associated with positivity of Mtb detection in BALF in an attempt to maximize the benefit of BALF collection for pulmonary TB suspects who undergo bronchoscopy. We found that age, cavity on chest radiographs, IGRAs results, and CD8^+^ T cell count are risk factors associated with positivity of BALF Mtb tests. By the stratified analysis, we determined that age, cavity on chest radiographs, IGRAs results, but not CD8^+^ T cell count, have an overlay effect in predicting positivity of Mtb tests in BALF. Specifically, a combination of age (≤ 35 years), IGRAs (+), cavity on chest radiographs had yielded a significantly high positivity (84.8%) of Mtb detection in BALF.

Among these factors, cavity on radiograph has the highest predictive value, which might be expected, as the cavities developed by erosion of caseation granulomas into bronchi, through which Mtb can be expelled [18]. In support of this, a previous report indicated numerous AFB are detected at the surface of cavities [19]. In the present study, age ≤ 35 years and positive IGRAs results were predictive factors for positive BALF results. Although IGRAs cannot reliably distinguish active TB from latent TB infection, we and other researchers have shown that the levels of IFN-γ determined by IGRAs are associated with sputum Mtb positivity in immune competent pulmonary TB patients [20], and the sensitivity of IGRAs is correlated with paucibacillary nature of the samples [21]. In line with these findings, here we found IGRAs alone has a predictive odds ratio of 3.743 (95% *CI:* 2.478–5.655) for Mtb positivity in BALF, due to the existence Non-TB patients in the BALF-negative TB suspects. We speculate that younger patients capable of mounting a robust cell-mediated immune response to pulmonary TB are more likely to develop cavitary lesions with positive sputum and BALF tests for Mtb.

## Conclusions

We found that age (**≤** 35 years), positive IGRA test and cavity on chest radiographs as risk factors associated with positivity of Mtb detection in BALF. Sputum negative pulmonary TB suspects who are under 35 years old, positive for the presence of pulmonary cavity and IGRA, should undergo bronchoscopy to collect BAFL for Mtb tests, as they have the highest possibility to get bacteriologically confirmation of TB.

## Additional file

Additional file 1: Multilingual abstracts in the six official working languages of the United Nations. (PDF 714 kb)

## Abbreviations

AFB: Acid fast bacilli
BALF: Bronchoalveolar lavage fluid
CDC: Center for Disease Control and Prevention
CIs: Confidence intervals
IGRAs: Interferon-gamma release assays
LPAs: Line probe assays
LTBI: Latent TB infection
MDR-TB: Multi-drug resistant TB
Mtb: *Mycobacterium tuberculosis*
NAAT: Nucleic acid amplification test
NTM: nontuberculous mycobacteria
ORs: Odds ratios
