# Supplementary material for: Indicators for prediction of Mycobacterium tuberculosis positivity detected with bronchoalveolar lavage fluid
Source: Infect Dis Poverty. 2018 Mar 24;7:22. doi: 10.1186/s40249-018-0403-x (PMC5868051; doi:10.1186/s40249-018-0403-x)
Supplement: Supplementary file 1 — Multilingual abstracts in the six official working languages of the United Nations. (PDF 714 kb) [file 40249_2018_403_MOESM1_ESM.pdf]

Translation of the abstract into the six official working languages of the United Nations

الجمع بين العمر، ومقايسة تحرير الانترفيرون-جاما، والتجويف الرئوي، يتنبأ بالكشف بإيجابية عالية عن جرثومة المتفطرة السلية في سائل غسل القصبات والأسناخ من المرضى المشتبه بإصابتهم بالسل سلبيي البلغم

شي ليو، شينغ-فانغ هوى، لي غاو، غو-فانغ دنغ، مينغ-شيا تشانغ، تشون-يي دنغ، تاو-شنغ يي، تشيان-تينغ يانغ، بو-بينغ تشو، تشى-هوا ون، هاي-يينغ ليو ، هاردي كورنفيلد، شين-تشون تشن

الملخص
الخلفية: لا يزال تشخيص السل الرئوي النشط يشكل تحدياً في العيادة، وخاصة بالنسبة للسل الرئوي السلبي البلغم (فحص البلغم لم يكشف وجود جرثومة المتفطرة السلية). سائل غسل القصبات والأسناخ لديه حساسية أعلى من البلغم للكشف عن المتفطرة السلية. ومع ذلك، فإن تنظير القصبات هو إجراء مكلف ويخترق الجسم وغير مناسب لكافة المرضى. في هذه الدراسة، نهدف إلى استكشاف مؤشر يُمكن استخدامه لتحسين اختيار تنظير القصبات إلى أبعد حد ممكن، حتى يتسنى لمرضى السل الاستفادة منه بشكل أكبر.
الطريقة: تم اختيار ما مجموعه 1539 من المشتبه بإصابتهم بمرض السل الرئوي سلبيي البلغم ممن خضعوا لتنظير القصبات من أجل التقييم. تمت مقارنة حساسية ونوعية ودقة الكشف عن جرثومة المتفطرة السلية في كل من البلغم وسائل غسل القصبات والأسناخ. تم استخدام نسبة الأرجحية ومستويات الثقة بنسبة 95٪ لتقييم المتغيرات التي ترتبط مع البلغم والذي كشف فحصها عن وجود العصيات الصامدة للحمض في مزرعة جرثومة المتفطرة السلية، واختبار تضخيم الحمض النووي لسائل غسل القصبات والأسناخ عند المشتبه بإصابتهم بمرض السل الرئوي سلبيي البلغم وعند المشتبه بإصابتهم بمرض السل ممن لا يفرزون البلغم.
النتائج: سائل غسل القصبات والأسناخ لديه حساسية تصل إلى (63.4 %) وهي أعلى بكثير من البلغم (43.5 %) للكشف عن جرثومة المتفطرة السلية عن طريق مزرعة جرثومة المتفطرة السلية، واختبار تضخيم الحمض النووي. كما تصل نسبة المشتبه بإصابتهم بمرض السل الرئوي سلبيي البلغم إلى 19.7 % من (620/122) وإلى 40.0 % من (408/163) من المشتبه بإصابتهم بالسل الرئوي ممن لا يفرزون البلغم ممن أظهروا نتائج بكتريولوجية إيجابية في سائل غسل القصبات والأسناخ. ومن بين المشتبه بإصابتهم بمرض السل الرئوي سلبي البلغم وممن لا يفرزون البلغم، فإن إيجابية الكشف عن جرثومة المتفطرة السلية في سائل غسل القصبات والأسناخ يرتبط بالعمر الأصغر وبوجود تجاويف رئوية ونتيجة إيجابية لفحص مقايسة تحرير الانترفيرون-جاما. المرضى سلبيي البلغم تحت سن 35 سنة مع نتيجة إيجابية لفحص مقايسة تحرير الانترفيرون-جاما ولديهم تجويف رئوي قد أظهروا إيجابية بنسبة 84.8 % لجرثومة المتفطرة السلية في سائل غسل القصبات والأسناخ.
الاستنتاجات: أشارت دراستنا إلى أن الجمع بين العمر، ووجود تجويف رئوي، ونتيجة فحص مقايسة تحريرالانترفيرون-جاما مفيد للتنبؤ بإيجابية الكشف عن جرثومة المتفطرة السلية في سائل غسل القصبات والأسناخ عند المشتبه بإصابتهم بمرض السل الرئوي سلبيي البلغم وممن لا يفرزون البلغم. أولئك الذين تقل أعمارهم عن 35 سنة، ولديهم تجويف رئوي ونتيجة ايجابية لفحص مقايسة تحريرالانترفيرون-جاما، يجب أن يخضعوا للتنظير القصبي لتجميع سائل غسل القصبات والأسناخ لاختبارات الكشف عن جرثومة المتفطرة السلية، لأن لديهم احتمال أكبر للحصول على تأكيد بكتيريولوجي بإصابتهم بالسل.

Translated from English version into Arabic by doctorammar, proofread by Manale Elewah, through

TRANSLATORS WITHOUT BORDERS

联合年龄、γ 干扰素释放试验和肺部空洞可预测痰菌阴疑似者肺泡灌洗液的结核菌检出情况

刘曦，侯杏芳，高磊，邓国防，张明霞，邓群益，叶涛生，杨倩婷，周伯平，温志华，刘海鹰，Hardy Kornfeld，陈心春*

摘要
引言：活动性肺结核，尤其是痰菌阴性肺结核的诊断在临床上仍然面临巨大的挑战。在支气管肺泡灌洗液中检出结核分枝杆菌的敏感度比痰液更高。然而，支气管镜为侵入性操作，且费用较高，并不适于广泛使用。本研究将探索哪些指标可能用于优化支气管镜的选择，使得肺结核患者可以更大程度地从中获益。
方法：本研究共纳入 1539 例痰涂片抗酸杆菌阴性的肺结核疑似者，比较了在痰和肺泡灌洗液这两种标本中检出结核分枝杆菌的敏感度和特异性。在痰结核菌阴性及无痰的肺结核疑似者中，我们采用比值比和 95% 置信区间来分析不同变量与在支气管肺泡灌洗液（抗酸染色、结核菌培养和核酸检测）获得阳性结果的相关性。
结果：支气管肺泡灌洗液的结核菌培养和核酸检测阳性率（63.4%）显著高于痰液（43.5%）。共有 19.7%（122/620）痰结核菌阴性及 40.0%（163/408）无痰的肺结核疑似者在支气管肺泡灌洗液中获得阳性结果。在痰结核菌阴性及无痰的肺结核疑似者中，支气管肺泡灌洗液获得阳性结果与年龄、肺部空洞或 γ 干扰素

释放试验阳性相关。有 84.8%的年龄≤35 岁，肺部存在空洞且γ干扰素释放试验阳性的痰菌阴性疑似者，支气管肺泡灌洗液结核菌检测为阳性。
结论：我们的研究提示，在痰结核菌阴性及无痰的结核疑似者中，联合年龄、肺部空洞及γ干扰素释放试验的结果有利于预测是否可从肺泡灌洗液中检测结核菌。其中≤35 岁、肺部存在空洞且γ干扰素释放试验阳性者，检出结核菌确诊肺结核的可能性最大，建议进行支气管镜采集肺泡灌洗液完善结核菌检查。

Translated from English version into Chinese by Xin-Chun Chen

Association de l'âge, de tests de libération d'interféron gamma et de cavités pulmonaires pour déterminer efficacement la positivité de la détection de *Mycobacterium tuberculosis* dans le liquide de lavage broncho-alvéolaire des cas soupçonnés dont les frottis sont négatifs

Xi Liu, Xing-Fang Hou, Lei Gao, Guo-Fang Deng, Ming-Xia Zhang, Qun-Yi Deng, Tao-Sheng Ye, Qian-Ting Yang, Bo-Ping Zhou, Zhi-Hua Wen, Hai-Ying Liu, Hardy Kornfeld, Xin-Chun Chen

Résumé
Contexte: diagnostiquer la tuberculose (TB) pulmonaire active demeure un défi en clinique, particulièrement pour les cas dont les tests sur les expectorations sont négatifs. Le liquide provenant du lavage broncho-alvéolaire (LLBA) est plus sensible que les expectorations en ce qui concerne la détection de *Mycobacterium tuberculosis* (Mtb). Cependant, la bronchoscopie est invasive, onéreuse et ne convient pas à tous les patients. Cette étude a pour but d'identifier les indicateurs pouvant être utilisés pour optimiser la bronchoscopie de façon à ce que les patients atteints de tuberculose en tirent le meilleur parti.
Méthodologie: un total de 1 539 cas soupçonnés de tuberculose pulmonaire dont les résultats au frottis sont négatifs mais qui ont subi une bronchoscopie ont participé à cette évaluation. La sensibilité, la spécificité et la précision de la détection de Mtb dans les expectorations et le LLBA ont été comparées. Des rapports de cotes et des intervalles de confiance à 95 % ont permis d'évaluer les variables associées aux bacilles acidorésistants positifs aux frottis, à la culture de Mtb et au test d'amplification des acides nucléiques (TAAN) dans le LLBA des cas soupçonnés de tuberculose pulmonaire ne produisant pas d'expectoration et de ceux dont les résultats aux frottis sont négatifs.
Résultats: la sensibilité du LLBA est nettement plus élevée (63,4 %) que celle des expectorations (43,5 %) pour la détection de Mtb dans les cultures et le TAAN. Pour 19,7 % (122/620) des cas soupçonnésdont les résultats aux frottis sont négatifs et 40,0 % (163/408) de ceux ne produisant pas d'expectoration, les résultats bactériologiques du LLBA se sont avérés positifs. Parmi les cas soupçonnés de tuberculose pulmonaire ne produisant pas d'expectoration et ceux dont les résultats aux frottis étaient négatifs, la capacité de détection de Mtb dans le LLBA est associée à un âge jeune, à la présence de cavités pulmonaires et à un résultat positif au test de libération d'interféron gamma (TLIG). Le taux de positivité à Mtb dans le LLBA pour les patients âgés de moins de 35 ans dont les résultats aux frottis se sont révélés négatifs, dont le TLIG était positif et qui présentaient des cavités pulmonaires s'élevait à 84,8 %.
Conclusions: notre étude a démontré que l'association de l'âge, de la présence de cavités pulmonaires et des résultats du TLIG permet de déterminer la positivité de la détection de Mtb dans le LLBA chez les cas soupçonnés de tuberculose pulmonaire ne produisant pas d'expectoration et chez ceux dont les résultats aux frottis étaient négatifs. Les individus de moins de 35 ans présentant des cavités pulmonaires et dont les résultats au TLIG sont positifs devraient se soumettre à une bronchoscopie dans le but de collecter du LLBA à des fins de test de présence de Mtb puisque la possibilité de confirmer, au niveau bactériologique, une TB est plus élevée chez cette population.

Translated from English version into French by Frank and Veronique Haour, through
TRANSLATORS
WITHOUT BORDERS

Сочетание возраста, результатов основанного на высвобождении гамма-интерферона анализа и наличие полости в ткани легких прогнозируют высокую вероятность обнаружения *микобактерий*

***туберкулеза* в жидкости бронхоальвеолярного лаважа у пациентов с отрицательными результатами анализа мокроты.**

Си Лю, Син-Фан Хоу, Лэй Гао, Го-Фан Дэн, Мин-Ся Чзан, Цюнь-И Дэн, Тао-Шен Е, Цянь-Тин Ян, Бо-Пин Чжоу, Чжи-Хуа Вэнь, Хай-Ин Лю, Харди Корнфельд, Синь-Чунь Чэнь

Аннотация
Справочная информация: Диагностика активного туберкулеза легких (ТБ) остается важной клинической проблемой, особенно в отношении туберкулеза легких у пациентов с отрицательными результатами анализа мокроты. Жидкость бронхоальвеолярного лаважа (БАЛ) имеет более высокую чувствительность по сравнению с мокротой для выявления *микобактерий туберкулеза* (МБТ). Однако бронхоскопия является инвазивной и дорогостоящей процедурой, поэтому метод не подходит для всех пациентов. В этом исследовании мы стремимся изучить, какой индикатор можно использовать с целью оптимизации выбора бронхоскопии с максимальной пользой для больных туберкулезом.
Методы: Оценку проводили у 1539 пациентов с подозрением на туберкулез легких, имеющих отрицательные результаты анализа мокроты; всем пациентам проводили бронхоскопию. Сравнивали чувствительность, специфичность и точность обнаружения МБТ в мазках мокроты с такими же показателями в жидкости БАЛ. Соотношения рисков и доверительный интервал 95% использовались для оценки переменных, связанных с положительным результатом на кислотоустойчивые бациллы в мазке, бактериальным посевом МБТ и методом амплификации нуклеиновых кислот жидкости БАЛ у пациентов с подозрением на туберкулез легких с отрицательными результатами анализа мокроты и без выделения мокроты.
Результаты: БАЛ имеет значительно более высокую чувствительность (63,4%), чем мазок мокроты (43,5%) для обнаружения МБТ при помощи бактериального посева и метода амплификации нуклеиновых кислот. У 19,7% (122/620) пациентов с отрицательными результатами анализа мокроты и 40,0% (163/408) пациентов без выделения мокроты были положительные результаты бактериологического анализа БАЛ. Среди пациентов с подозрением на туберкулез легких с отрицательными результатами анализа мокроты и без выделения мокроты положительные результаты обнаружения МБТ в жидкости БАЛ ассоциируются с более молодым возрастом, наличием легочных полостей и положительным результатом анализа, основанного на высвобождении гамма-интерферона (IGRA). У пациентов с отрицательными результатами анализа мокроты в возрасте до 35 лет, но при наличии положительного результата анализа IGRA и легочной полости, в 84,8% случаев были обнаружены МБТ в жидкости БАЛ.
Заключение: Наше исследование показало, что сочетание возраста, наличия полости в ткани легких и результата анализа IGRA, помогает прогнозировать обнаружение МБТ в жидкости БАЛ у пациентов с подозрением на туберкулез легких, если у них отрицательные результаты анализа мокроты, и они не выделяют мокроту. Пациенты моложе 35 лет, имеющие полость в ткани легких и положительный результат анализа IGRA, должны пройти бронхоскопию для сбора жидкости БАЛ с целью проведения анализов на МБТ, так как такие пациенты имеют самую большую вероятность бактериологического подтверждения туберкулеза.

Translated from English version into Russian by Oksana Rozhko and Ann Nosova, through

TRANSLATORS
WITHOUT BORDERS

La combinación de edad, ensayo de liberación de interferón-gamma y la cavidad pulmonar predicen una alta positividad de *detección de Mycobacterium tuberculosis* en el líquido de lavado broncoalveolar de sospechosos de esputo negativo

Xi Liu, Xing-Fang Hou, Lei Gao, Guo-Fang Deng, Ming-Xia Zhang, Qun-Yi Deng, Tao-Sheng Ye, Qian-Ting Yang, Bo-Ping Zhou, Zhi-Hua Wen, Hai-Ying Liu, Hardy Kornfeld, Xin-Chun Chen

Resumen

Antecedentes: El diagnóstico de tuberculosis pulmonar activa (TB) sigue siendo un desafío en la clínica, especialmente para la tuberculosis pulmonar negativa de esputo. El líquido de lavado broncoalveolar (BALF) tiene una mayor sensibilidad que el esputo para la detección de *Mycobacterium tuberculosis* (Mtb). No obstante, la broncoscopia es invasiva y costosa, y no es adecuada para todos los pacientes. En este estudio, nuestro objetivo es explorar qué indicador se puede utilizar para optimizar la elección de la broncoscopia, para que los pacientes con TB puedan obtener un mayor beneficio de ella.
Métodos: Se reclutó un total de 1539 pacientes con tuberculosis pulmonar con frotis de esputo negativo que se sometieron a una broncoscopia para su evaluación. Se comparó la sensibilidad, especificidad y precisión de la detección de Mtb en esputo y BALF. Se usaron odds ratios e intervalos de confianza del 95% para evaluar variables asociadas con frotis positivos de bacilos acidorresistentes (AFB), cultivo de Mtb y prueba de amplificación de ácidos nucleicos (NAAT) de BALF en sospechosos de tuberculosis pulmonar productores de esputo negativos y no productores de esputo.
Resultados: El BALF tiene una sensibilidad significativamente mayor (63.4%) que el esputo (43.5%) para la detección de Mtb por cultivo y NAAT. 19,7% (122/620) esputo-negativo y 40,0% (163/408) sospechosos no productores de esputo tuvieron resultados bacteriológicos positivos en BALF. Entre los sospechosos de tuberculosis pulmonar con esputo negativo y sin esputo, la positividad de la detección de Mtb en BALF se asocia con una edad más joven, la presencia de cavidades pulmonares y un resultado positivo del ensayo de liberación de interferón gamma (IGRA). Los pacientes con esputo negativo menores de 35 años con IGRA positivo y cavidad pulmonar tenían un 84,8% de positividad de Mtb en BALF.
Conclusiones: Nuestro estudio indicó que la combinación de edad, la presencia de cavidad pulmonar y el resultado de IGRA es útil para predecir la positividad de detección de Mtb en BALF entre sospechosos de tuberculosis pulmonar productores de esputo negativos y no esputo. Los menores de 35 años, positivos para la presencia de cavidad pulmonar e IGRA, deben someterse a una broncoscopia para recolectar BAFL para las pruebas de Mtb, ya que tienen la posibilidad mayor de obtener la confirmación bacteriológica de la TB.

Translated from English version into Spanish by Sylvia and Susana Rosselli, through

TRANSLATORS
WITHOUT BORDERS
